# Levels of toxic and trace metals in the breast milk of lactating mothers in Abeokuta, Ogun State, Nigeria

**DOI:** 10.1016/j.toxrep.2023.08.001

**Published:** 2023-08-09

**Authors:** Olanrewaju Olujimi, Sulaimon Ajakore, Damilola Abuganloye, Toyin Arowolo, Oliver Steiner, Walter Goessler, Taofeek Towolawi

**Affiliations:** a Department of Environmental Management and Toxicology, Federal University of Agriculture, P.M.B. 2240 Abeokuta, Ogun State, Nigeria; b Institute for Chemistry, Department of Analytical Chemistry, Karl-Franzens University, Universitaesplatz 1, 8010 Graz, Austria; c Department of Environmental Health Science, College of Basic Medical and Health Sciences, Fountain University Osogbo, Osun State, Nigeria

**Keywords:** Human breast milk, Metal exposure, ICP-MS, Allergic reactions, Dysfunction, Pollution

## Abstract

**Background and objectives:**

Breast milk is an essential source of nutrients and energy for infants. The study analyzed for the levels of essential, toxic and rare earth elements in the breast milk of lactating mothers within Abeokuta metropolis.

**Materials and methods:**

Thirty-seven (37) breast milk samples were collected with consents of lactating mothers at Ogun State General Hospital in Abeokuta. The samples were digested using standard method and analyzed for essential, toxic and rare earth elements using Inductively Coupled Plasma Mass Spectrometer (ICP-MS). The data were subjected to descriptive analysis.

**Results:**

The results showed higher concentrations of toxic elements than essential elements in the breast milk of lactating mothers, where five toxic metals: Ag, Ti, V, Pb and Ba were observed to be present in 11, 14, 15, 17 and 23 breast milk samples respectively. Two essential (P and S) and two toxic (Cd and Hg (except sample 19)) elements were observed to be present in all the breast milk samples. Rare Earth Elements (except Sr, U and Rb) were below the detection limit of the instrument. Though three breast milk samples (12, 14 and 17) were observed safe, they contained two toxic (Cd and Hg) and a rare earth trace (Rb) element.

**Conclusion:**

It could be concluded that despite the inherent benefits of human breast milk with essential elements to the infants, it can still be a source of toxic and trace earth metals contamination.

## Highlights

• Breastmilk were analyzed from lactating mothers.
• Ag, Ti, V, Pb and Ba and Rare earth element Sr, U and Rb were detected in breastmilk samples.
• Metal detection a reflection of possible food consumption and residential areas.

## Graphical Abstract

**Figure:**
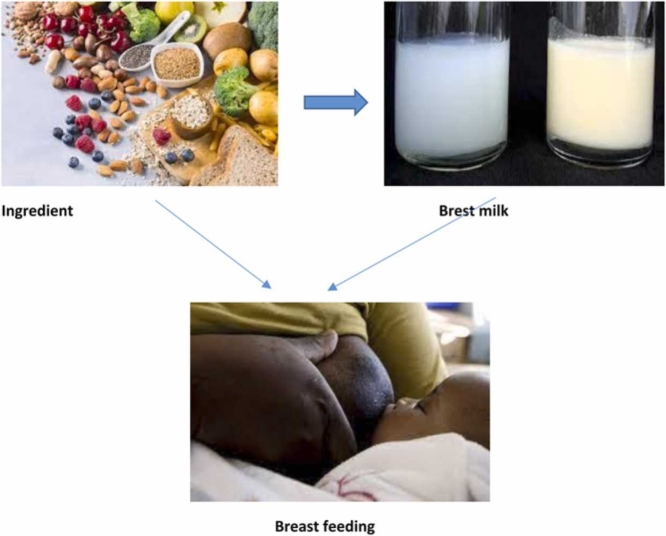

## 1 Introduction

Breast milk is the main source of food for infants and provides proteins, fats, carbohydrates and essential elements for normal body growth [1]. The World Health Organization (WHO) recommends exclusive breastfeeding for the first six months of life. Supplemented breastfeeding is recommended until at least age two and for as long as the mother and child wish [2]. This is because breast milk comprises of micro- and macro- nutrients that infant requires for healthy growth. It also has protective proteins, which are the antibodies and enzymes which antagonize infections in infants [3], [4]. Breast milk contains functional proteins, which bind with metal ions and become efficient or inefficient metalloproteinase in the human body fluids [5]. Components such as lactoferrin, lysozyme and α-lactalbumin of mother's milk guard the newborns against injurious environmental elements thereby enhancing the defense mechanisms of body. The components also stimulate the immune system development [6] and reduce the risk of allergies and autoimmune diseases [7].

Breastfeeding offers health benefits to child even after infancy; these benefits include a 73 % decrease risk of sudden infant death syndrome, increased intelligence, decrease likelihood of contracting middle ear infections, cold and flu resistance, decrease in the risk of childhood leukemia, lowers childhood onset diabetes, decreases risk of asthma and eczema, decreases dental problems and risk of obesity later in life [2]. Breastfeeding also provides health benefits to the mother by assisting the uterus and mother weight returning to pre-pregnancy size. It also reduces the risk of breast cancer later in life. Environmental pollutants such as metals had been on the increase with increase population due to industrialization and urbanization [8]. Pregnant women and nursing mothers are exposed to such metal pollutants on daily basis. Metallic pollutants as a result of increasing industrialization have penetrated into all spheres of life [9]. Studies had claimed that breast milk can be a pathway of maternal excretion of toxic elements and other contaminants with probable harmful effect on nervous system of infants [10], [11] Accumulation of toxicants in the body systems is generally higher for newborns and infants than the adults, who are less susceptible and sensitive to the danger of toxic substances exposure [12].

Dichlorodiphenyltrichloroethane (DDT) was the first toxic chemical found in human breast milk [13]. Heavy metals and other contaminants have been reported in the breast milk of lactating mothers [10]. One reason for seldom monitoring of heavy metals such Pb, Hg and Cd in breast milk is that their concentrations are only about 20 % of their amount in maternal blood. This is associated to their low lipophilic and high erythrocytes adsorption. Heavy metals are also present in either inorganic or organometallics form [14]. Effort had been made to measure transfer factors of some selected metals from food into breast milk samples; the results had it that the values varied across the lactating mothers [15]. Gapped variations in breast milk elemental concentrations are possibly influenced by factors that are not only limited to environmental, geographic, nutritional, occupational and individual lifestyles but also include both analytical and instrumental factors [16], [17]. As maternal stored toxic metals move into break milk during pregnancy and lactation, breast feeding infants may be ingesting the toxic metals [18]. This research thus assessed breast milk of nursing mothers for essential, toxic and other metals so as evaluate probable health risks the infants are posed from being breastfed.

## 2 Materials and methods

### 2.1 Donor-mother information

Questionnaire was administered to the donor-mother to gather information that ranged from age, occupation, residential area, smoking and dietary habits and personal hygiene. Questions on diet include consumption of vegetables, fruits, fish, meat, mushrooms and cereals.

### 2.2 Samples collection and preservation

Breast milk samples were collected from lactating mothers who voluntarily participated after consenting to the objectives of the study. Thirty-seven (37) cohorts were revisited and their breasts were cleaned with cotton wool and distilled water to avoid contamination with dust particles that might be loaded with heavy metals before manual breast samples collection. Each donor-mother expressed her breast gently herself while collecting the breast milk sample. The first few drops of the breast milk were used to rinse the samples containers and about 10 mL of breast sample was collected from each lactating mother into the container and labeled. The samples were stored at − 20 °C in the laboratory before analysis.

### 2.3 Sample preparation and digestion

Five (5) mL of the sample was transferred into a conical flask, followed with addition of 4 mL HNO_3_ and 2 mL H_2_O_2_. The conical flask was then heated gently on a hot plate for 5 min. After cooling, the digested sample was transferred to a 50 mL volumetric flask. Samples were analyzed at the Institute of Chemistry, Department of Analytical Chemistry, University of Graz, Graz, Austria using ICP-MS (7500ce. Agilent). Samples were analyzed for toxic metals, essential and rare earth elements.

#### 2.3.1 Ethical approval

This study was approved by the Director of Health Services, Federal University of Agriculture, Abeokuta and the Ethics Committee of the Ogun State Hospital Management Board. All procedures performed in the study were in accordance with the ethical standards of the committee and with the 1964 Helsinki declaration and its later amendments. Informed consents (verbal) were obtained from all participants (donor mothers) before taking part in the study.

## 3 Results and discussion

### 3.1 Metals in the breast milk

#### 3.1.1 Essential elements in breast milk

Twelve essential elements (Li, B, Na, P, S, Ca, Cr, Mn, Fe, Co, Cu and Zn) were determined in the breast milk. From Table 1, the level of Li was observed to range from < LOD to 120 ± 4 with a pool mean of 28.86 ± 39.66 µg/L. The B concentration (µg/ L) ranged from < LOD to 1710 ± 35 µg/L and its mean level was 428.11 ± 590.75 µg/L. The value detected in this study was observed to be higher than the values reported for B in breast milk samples of Swedish (24 μg/L) and US women (30 μg/L) [19]. The levels of B were reported unchanged in the course of lactation; it was so assumed that milk B levels could have been controlled in the mammary gland homeostasis [20]. Levels of Na (µg/L) ranged from < LOD to 238 ± 22 mg/L with a pool mean level of 38.86 ± 62.75 mg/L. The detected levels in this study were higher than the daily intake prescribed by the United States Department of Health and Human Services in USA [21]. Phosphorus level was observed to range from 15 ± 3 to 258 ± 13 mg/L with a pool mean level of 109 ± 60 mg/L. The level of S ranged from 8.4 ± 2.3 to 157 ± 11 mg/L with a pool mean level of 72.3 ± 37.2 mg/L. Concentrations of Ca were observed to range from < LOD to 2972 ± 32 mg/L with an average value of 532 ± 534 mg/L. The concentration of Cr and Mn was respectively determined to range from < LOD to 940 ± 7 µg/L with a pool mean value of 89.95 ± 154.1 and < LOD and 1160 ± 12 µg/L with an average value of 187.57 ± 319.55 µg/L. The determined value of Cr in the current study was found to be greater than previous studies in Saudi Arabia [22] and Nigeria [23]. The levels of Fe were observed to range from < LOD to 6980 ± 12 µg/L with a mean value of 1732 ± 1993 µg/L while Co concentration ranged from < LOD to 44 ± 7 µg/L with a mean value of 2.14 ± 7.73 µg/L. The determined value of Fe in the current study was found to be higher than previous study in Saudi Arabia [22] but lower than the studies in Greece [24], Nigeria [23] and Turkey [25]. Iron is essential for oxygen and electron transfer in human body. Cobalt is a part of Cyanocobalamin (Vitamin B_12_) which is necessary for productions of DNA and needed to enhance levels of hemoglobin. Cobalt is a primary cause of contact dermatitis and concern regards its being potential carcinogen has been reported [26].

**Table 1:** Concentrations of essential elements in breast milk.

Subjects	Li (µg/L)	B (µg/L)	Na (mg/L)	P (mg/L)	S (mg/L)	Ca (mg/L)	Cr (µg/L)	Mn (µg/L)	Fe (µg/L)	Co (µg/L)	Cu (µg/L)	Zn (µg/L)
1	10 ± 2	LOD	88.6 ± 12	25.8 ± 46	151 ± 11	1157 ± 23	940 ± 7	1040 ± 12	6980 ± 12	LOD	540 ± 11	LOD
2	LOD	LOD	LOD	191 ± 11	88 ± 13	477 ± 22	36 ± 8	230 ± 6	1190 ± 19	LOD	180 ± 3	LOD
3	6 ± 1	810 ± 32	53 ± 5	201 ± 23	139 ± 11	713 ± 12	140 ± 5	520 ± 4	3010 ± 17	LOD	320 ± 6	2690 ± 12
4	86 ± 12	1510 ± 11	136 ± 4	130 ± 15	102 ± 23	352 ± 15	76 ± 3	LOD	640 ± 13	LOD	200 ± 12	LOD
5	5 ± 1	130 ± 4	52.5 ± 3	122 ± 12	86 ± 7	853 ± 19	140 ± 3	720 ± 11	2480 ± 17	LOD	300 ± 9	LOD
6	LOD	LOD	LOD±	127 ± 9	56 ± 4	587 ± 21	LOD	340 ± 15	990 ± 12	LOD	140 ± 9	LOD
7	3 ± 1	240 ± 6	105 ± 6	248 ± 13	157 ± 11	415 ± 13	180 ± 3	130 ± 17	LOD	LOD	550 ± 11	4310 ± 12
8	3 ± 1	LOD	170 ± 9	146 ± 21	145 ± 13	485 ± 16	35 ± 5	520 ± 11	6850 ± 13	LOD	370 ± 9	1910 ± 5
9	7 ± 2	230 ± 9	37.9 ± 11	155 ± 19	99 ± 12	1129 ± 19	130 ± 4	1040 ± 9	6450 ± 21	LOD	370 ± 5	1410 ± 7
10	4 ± 1	200 ± 12	LOD	148 ± 11	101 ± 9	624 ± 21	43 ± 9	430 ± 6	4090 ± 8	LOD	170 ± 11	LOD
11	LOD	LOD	LOD	93 ± 5	45 ± 23	438 ± 22	200 ± 11	LOD	LOD	LOD	180 ± 15	LOD
12	LOD	LOD	LOD	41 ± 3	17 ± 6	LOD	LOD	LOD	LOD	LOD	89 ± 15	LOD
13	LOD	LOD	233 ± 14	46 ± 7	84 ± 21	347 ± 23	86 ± 7	LOD	LOD	LOD	120 ± 21	LOD
14	LOD	LOD	LOD	80 ± 4	39 ± 11	LOD	LOD	LOD	LOD	LOD	110 ± 11	LOD
15	1 ± 0.2	LOD	LOD	36 ± 9	38 ± 5	1095 ± 12	54 ± 9	250 ± 7	1910 ± 9	LOD	88 ± 22	LOD
16	LOD	LOD	LOD	22 ± 2	8.4 ± 2.3	LOD	LOD	LOD	LOD	LOD	LOD	LOD
17	LOD	LOD	LOD	83 ± 4	57 ± 5	61 ± 11	LOD	LOD	LOD	LOD	110 ±	LOD
18	7 ± 2	LOD	LOD	116 ± 2	69 ± 13	2972 ± 32	150 ± 5	1160 ± 12	5270 ± 12	4 ± 1	200 ± 17	1410 ± 12
19	LOD	LOD	LOD	22 ± 1	13 ± 8	541 ± 19	LOD	LOD	LOD	LOD	LOD	LOD
20	2 ± 0.3	140 ± 12	31 ± 3	140 ± 11	90 ± 12	629 ± 17	120 ± 8	LOD	2280 ± 22	LOD	300 ± 21	1700 ± 15
21	LOD	LOD	70 ± 12	63 ± 6	48 ± 11	42 ± 11	LOD	LOD	1620 ± 34	LOD	100 ± 16	LOD
22	4 ± 2	LOD	LOD	132 ± 3	73 ± 5	692 ± 21	160 ± 4	140 ± 9	5010 ± 13	LOD	160 ± 15	LOD
23	27 ± 7	LOD	LOD	33 ± 5	28 ± 1	LOD	LOD	LOD	720 ± 19	16 ± 3	29 ± 2	LOD
24	29 ± 4	LOD	44 ± 7	152 ± 9	81 ± 4	167 ± 3	LOD	LOD	LOD	13 ± 2	120 ± 12	LOD
25	LOD	LOD	LOD	115 ± 11	67 ± 5	935 ± 5	79 ± 2	150 ± 6	1870 ± 23	LOD	140 ± 14	LOD
26	80 ± 9	1030 ± 22	LOD	43 ± 5	33 ± 7	399 ± 7	100 ± 3	LOD	LOD	LOD	77 ± 11	LOD
27	91 ± 13	1220 ± 12	37.8 ± 13	15 ± 3	43 ± 11	512 ± 3	100 ± 5	LOD	680 ± 11	LOD	39 ± 7	LOD
28	LOD	LOD	LOD	120 ± 12	70 ± 19	608 ± 7	59 ± 2	LOD	860 ± 43	LOD	82 ± 6	LOD
29	110 ± 9	1710 ± 35	78 ± 12	83 ± 9	70 ± 13	518 ± 11	60 ± 4	LOD	LOD	LOD	49 ± 5	920 ± 9
30	LOD	LOD	LOD	83 ± 4	68 ± 15	866 ± 13	140 ± 2	150 ± 13	1630 ± 23	LOD	170 ± 3	LOD
31	78 ± 6	1240 ± 55	238 ± 22	120 ± 12	114 ± 22	899 ± 5	100 ± 4	120 ± 12	1370 ± 11	LOD	200 ± 3	LOD
32	70 ± 3	1180 ± 43	LOD	37 ± 4	39 ± 2	LOD	100 ± 3	LOD	1160 ± 5	LOD	91 ± 4	LOD
33	89 ± 12	1290 ± 23	LOD	54 ± 9	30 ± 5	LOD	LOD	LOD	1850 ± 9	LOD	47 ± 8	LOD
34	120 ± 4	1430 ± 12	LOD	139 ± 12	66 ± 7	100 ± 8	LOD	LOD	1150 ± 23	2 ± 0	210 ± 9	LOD
35	99 ± 11	1650 ± 13	LOD	139 ± 23	75 ± 8	108 ± 9	LOD	LOD	960 ± 22	44 ± 7	180 ± 5	LOD
36	84 ± 5	1090 ± 15	40 ± 13	153 ± 13	96 ± 11	206 ± 21	LOD	LOD	1830 ± 16	LOD	210 ± 3	1700 ± 23
37	53 ± 3	740 ± 19	22 ± 2	128 ± 11	88 ± 13	771 ± 11	100 ± 7	LOD	1250 ± 11	LOD	160 ± 12	LOD

The levels Cu and Zn ranged respectively from < LOD to 550 ± 11 µg/L with an average value of 173 ± 127 µg/L and < LOD to 4310 ± 12 µg/L with mean value of 433.78 ± 958.89 µg/L. The determined mean value of Cu in the current study was found to be lower than previous studies in Saudi Arabia, Nigeria and Greece [22], [23], [24] but greater in other studies in Italy, Sweden and Poland [27], [28], [29]. The value of Zn in the current study was found to be lower than previous studies [24], [25] in Greece and Turkey but greater than values reported in Sweden, Saudi Arabia and Italy [22], [27], [28]. Zinc is used by the athletes to improve their performance and strength; it is also used in the production of toothpaste. Both Zn and Cu are integral parts of various enzymes, proteins and tissues. Their mobility is governed by metallothionein [30] and attachment is moderately to the same proteins such as lactalbumin in colostrum and transitional milk [31].

The levels of all the essential elements in this study exceeded the values determined in the study of Björklund [28]. Both P and S were detected in all the breast milk samples while Cu, Ca, Fe, Li and Cr were detected in 95 %, 84 %, 70 %, 65 % and 65 % of the samples. About 40 % of the samples contained B, Na and Mn while Co was only found in 13.5 % of all the breast milk samples analyzed in this study. The results from this study for Fe, Cu, Cr and Zn were higher than values reported in breast milk of lactating mothers in Eastern, Nigeria [32].

### 3.2 Toxic elements in breast milk

Examples of toxic elements include Ti, V, Ni, Cd, As, Cr, Sn, Hg, Pb, Al, Mn and Li. The contents of toxic metals in breast milk reflect the level of environmental pollution and the mother's diet [33], [34], [35], [36]. From Table 2, Ni levels were detected to range from < LOD to 130 ± 13 µg/L with a pool mean value of 10.89 ± 46.9 µg/L. The Ni mean value was lower than the values detected in previous studies [22], [37]. Only 4 out of 37 (11 %) breast milk samples contained Ni concentrations. Nickel is not only lethal to the reproductive and pulmonary systems but it is also a concerned toxic element to different systems in humans and hematotoxic, immunotoxic, neurotoxic, nephrotoxic, hepatotoxic, and carcinogenic mediator [38]. The most prominent deleterious clinical effect of Ni in man is an allergic skin response in those susceptible to nickel skin irritation [38].

**Table 2:** Concentrations (µg/ L) of toxic elements in breast milk.

Sub	Ti	V	Ni	Ag	Cd	As	Sn	Ba	Hg	Pb
1	200 ± 7	LOD	130 ± 13	11.5 ± 1.6	22 ± 2	48.9 ± 11.3	LOD	1750 ± 11	15.3 ± 2.3	400 ± 22
2	LOD	LOD	LOD	5.2 ± 0.4	9 ± 1	LOD	LOD	550 ± 21	13.4 ± 3.2	LOD
3	36 ± 3	LOD	LOD	6.8 ± 0.7	4.9 ± 1.4	LOD	LOD	940 ± 19	149 ± 19	LOD
4	LOD	LOD	20	LOD	71.6 ± 2.6	371 ±	LOD	1560 ± 22	11.8 ± 0.9	LOD
5	19 ± 2	LOD	100	24.1 ± 1.4	5.5 ± 1.3	LOD	LOD	1320 ± 14	7.4 ± 0.4	96 ± 4
6	LOD	LOD	LOD	LOD	40.3 ± 2.1	LOD	LOD	720 ± 8	9.3 ± 2.1	LOD
7	LOD	LOD	LOD	6.8 ± 2.1	3.9 ± 0.8	LOD	LOD	360 ± 4	13.7 ± 3.1	63 ± 7
8	122 ± 4	LOD	LOD	8.5 ± 2.3	63.1 ± 11.4	LOD	LOD	600 ± 9	11.4 ± 2.2	255 ± 11
9	88 ± 5	4.30 ± 0.5	70 ± 21	43.4 ± 1.1	35.7 ± 2.8	LOD	LOD	1720 ± 21	16.2 ± 4.3	289 ± 21
10	63 ± 7	LOD	LOD	8.6 ± 0.9	15.1 ± 1.7	LOD	LOD	910 ± 12	16.8 ± 2.2	47 ± 8
11	LOD	LOD	LOD	LOD	4.99 ± 1.09	LOD	LOD	LOD	6.4 ± 1.7	224 ± 3
12	LOD	LOD	LOD	9.5 ± 1.3	3.3 ± 0.1	LOD	LOD	LOD	3.9 ± 0.3	LOD
13	LOD	LOD	LOD	LOD	26.1 ± 2.2	LOD	LOD	LOD	12.6 ± 0.6	68 ± 7
14	LOD	LOD	LOD	LOD	13.2 ± 1.5	LOD	LOD	LOD	4.9 ± 1.1	LOD
15	LOD	18.5	LOD	LOD	71.8 ± 2.6	LOD	LOD	110 ± 11	3.2 ± 0.3	64 ± 3
16	LOD	LOD	LOD	LOD	85.5 ± 3.5	LOD	LOD	LOD	3.3 ± 0.1	LOD
17	LOD	LOD	LOD	LOD	30 ± 2	LOD	LOD	LOD	9.0 ± 2	LOD
18	50 ± 2	72.2 ± 3.2	LOD	LOD	181 ± 13	17.9 ± 2.9	22.7 ± 1.6	750 ± 22	8.6 ± 2.1	104 ± 11
19	LOD	5.4 ± 0.9	LOD	LOD	31.8 ± 0.6	LOD	LOD	LOD	LOD	LOD
20	25 ± 4	4.2 ± 0.6	LOD	LOD	198 ± 11	LOD	LOD	LOD	9.0 ± 3	67 ± 9
21	39 ± 5	LOD	LOD	LOD	48.1 ± 0.7	LOD	LOD	LOD	5.8 ± 0.9	LOD
22	110 ± 6	11.1 ± 1.5	LOD	10.6 ± 1.2	33.8 ± 1.3	LOD	LOD	LOD	11.7 ± 0.7	97 ± 5
23	LOD	LOD	LOD	LOD	3.9 ± 0.3	LOD	LOD	LOD	4.4 ± 0.3	LOD
24	LOD	LOD	LOD	LOD	9.4 ± 1.5	LOD	LOD	LOD	4.4 ± 0.2	57 ± 4
25	LOD	12.0 ± 2.0	LOD	LOD	63.7 ± 3.3	LOD	LOD	95 ± 21	7.2 ± 1.1	162 ± 6
26	LOD	LOD	LOD	LOD	49.8 ± 4.1	329 ± 22	LOD	1040 ± 19	6.7 ± 1.7	LOD
27	LOD	5 ± 1	LOD	8.7 ± 2.5	50.8 ± 6.3	356 ± 11	LOD	1300 ± 13	5.6 ± 2.2	LOD
28	LOD	4.7 ± 1.2	LOD	LOD	39.7 ± 3.4	LOD	LOD	LOD	6.0 ± 1.3	LOD
29	LOD	LOD	LOD	LOD	74 ± 12	367 ± 23	LOD	1650 ± 123	5.9 ± 1.1	LOD
30	LOD	13.9 ± 1.8	LOD	LOD	91.3 ± 13.1	LOD	LOD	LOD	7.6 ± 1.9	LOD
31	LOD	12 ± 2	LOD	LOD	87.9 ± 12.2	351 ± 21	LOD	1230 ± 22	7.3 ± 2.3	LOD
32	38 ± 3	LOD	LOD	LOD	6.9 ± 1.4	212 ± 22	LOD	900 ± 23	10.8 ± 3.6	LOD
33	42 ± 8	6.5 ± 1.7	LOD	LOD	46 ± 11	334 ± 32	LOD	1050 ± 19	5.7 ± 0.9	1110 ± 21
34	23 ± 2	4.4 ± 1.2	LOD	LOD	16 ± 4	500 ± 49	LOD	1310 ± 13	5.6 ± 2.1	LOD
35	LOD	4.2 ± 1.1	LOD	LOD	18.2 ± 1.6	505 ± 55	LOD	1250 ± 11	5.3 ± 1.2	533 ± 19
36	27 ± 2	LOD	LOD	LOD	36.5 ± 2.2	124 ± 11	LOD	840 ± 15	5.2 ± 1.4	398 ± 12
37	LOD	8.3 ± 1.2	LOD	LOD	44.2 ± 3.1	278 ± 13	LOD	840 ± 33	7.1 ± 2.1	LOD

The detected Cd levels ranged from 3.3 ± 0.1 to 198 ± 11 µg/L with its mean concentration 45.46 ± 43.4 µg/L exceeded the values previously reported [1], [11], [16], [21], [22], [24], [39], [40]. The authors observed that the levels of Cd are not only varied in foodstuff but also in individual intake considerably due to dietary habits. It is important to note that Cd was detected in all the breast milk samples. Generally, leafy vegetables are found to contain high levels of Cd at about 0.05–0.12 mg/kg [41]. Levels of As varied from < LOD to 505 ± 55 µg/L with its the mean value evaluated as 291.9 ± 152.9 µg/L which exceeded previous studies [21], [37], [40] reported values in Ghana, Turkey and Cyprus. The study showed that 13 out of 37 (35.13 %) breast milk samples contained As levels more than the mean concentration previously reported. The major sources of As contamination are usage or inhalation of arsenic containing compounds such as herbicides and pesticides. Levels of Hg in the breast milk samples varied from < LOD to 149 ± 19 µg/L with its mean pool concentration of 8.4 ± 3.9 µg/L. The latter was found to be below the previous reported values [11], [25], [39], [40] in Iran, Turkey, Saudi Arabia and Cyprus but greater than the results of Gundacker [34] and Yang [42] in Austria and China, respectively.

Levels of Pb varied from < LOD to 1110 ± 21 µg/L with its mean concentration: 237 ± 268.5 µg/L exceeded the values reported in nine countries: Turkey, Green, Sweden, Brazil, Italy, Saudi Arabia, Ghana, Iran and Cyprus. A high value of 1020 µg/L was reported in Turkey [37]; which is close to the result obtained in the current study (1110 µg/L). Lead was detected in 46 % of all the analyzed samples. Lead is the most recognized toxic environmental pollutant and accumulates in the skeleton especially bone marrow. Lead retards children’s growth by accumulating in the long bones resulting in bone damage [43], [44]. It is a neurotoxin and influencing behavioral abnormalities, retarding intelligence and mental development [45].

Human contact with As, Cd, Hg and Pb could result in neurotoxic and genotoxic effects and deleterious health problems [41], [46]. Abnormal infant development can irreversibly result from excessive exposure to these toxic metals [47]. Abnormal physical growth of foetuses, infants and/or older children had been observed to adversely result from low-level exposure of As, Cd, Hg and Pb [48], [49], [50], [51]. Low level of Cd is released into human breast milk because proteins that bind Cd in breast milk bind more to calcium because of the high amount of calcium released into human breast milk, thus there is competitive inhibition of the binding proteins of Cd [46], [52]. Organometallic form of mercury (methylmercury) binds to sulfhydryl groups where it is central nervous system toxicant while inorganic mercury is a nephrotoxicant [14]. The interactions of proteins in human breast milk with both Ca and Cd vary reasonably and so contribute to the inherent variation of toxic metals in breast milk [53].

The level of Al was below the detection limit of the instrument in all the breast milk samples and lower than the mean value previously reported [29]. Toxic metals are eliminated from the body of nursing mother through hair, breast milk and urine [54], [55]. Some detected diseases such as intrauterine growth retardation, aspirated syndrome, respiratory distress syndrome, edematous syndrome were traced to metal concentrations in human milk samples fed the newborns (preterm and full term infants) [56]. It was however observed that the breast milk contains a large amount of proteins and antioxidants [57], [58], [59], which have detoxifying effect against heavy metals [55].

### 3.3 Rare earth elements in the breast milk

Trace elements analyzed in the current study included Ga, Rb, Sr, Te, Bi, U and Tl. The concentrations of Ga, Te, Bi and Tl were below limits of detection of the analytical instrument (Table 3). Levels of Rb, Sr and U were observed to respectively vary from 118 to 1510 µg/L with mean concentration value of 563. 5 ± 325.1 µg/L, < LOD to 2200 µg/L with mean concentration value of 307.4 ± 403.7 µg/L, and < LOD to 11.2 µg/L with mean concentration value of 3.5 ± 0.3 µg/L. The mean concentration values of both Rb: 563.5 ± 325.1 µg/L and Sr: 307.4 ± 403.7 µg/L in the current study were respectively lower and higher than the values reported in previous studies (R: 714 and 690) [60] and (Sr: 33 and 31) [28]. The respective authors observed that low variation between Rb and Sr breast milk concentrations in the women showed there was little effect from maternal intake of both Rb and Sr from the environmental exposure. However, the value of U in the current study (3.5 ± 0.3 µg/L) was greater than the value (0.42 ± 0.40 µg/L) previously reported [28].

**Table 3:** Concentrations (µg/ L) of Trace elements in breast milk.

Sub	Rb	Sr	U
1	1310 ± 21	750 ± 13	11.2 ± 1.4
2	527 ± 11	170 ± 9	4.4 ± 0.8
3	974 ± 19	390 ± 32	6.5 ± 1.3
4	786 ± 12	140 ± 12	2.9 ± 0.4
5	545 ± 13	520 ± 23	8.5 ± 1.7
6	394 ± 9	250 ± 22	5.7 ± 0.6
7	1510 ± 22	100 ± 9	3 ± 1
8	612 ± 13	250 ± 21	4 ± 1
9	676 ± 14	710 ± 14	10.8 ± 0.3
10	1110 ± 11	320 ± 19	5.8 ± 0.9
11	432 ± 13	180 ± 17	1.8 ± 0.3
12	184 ± 9	LOD	LOD
13	264 ± 12	100 ± 10	1.4 ± 0.3
14	364 ± 17	LOD	LOD
15	252 ± 5	706 ± 10	4.4 ± 1.2
16	145 ± 4	LOD	LOD
17	495 ± 11	LOD	LOD
18	541 ± 12	2200 ± 210	10 ± 3
19	118 ± 15	380 ± 15	2.2 ± 0.2
20	653 ± 12	370 ± 23	2.1 ± 0.1
21	415 ± 18	LOD	LOD
22	561 ± 17	350 ± 16	2.9 ± 0.4
23	150 ± 7	LOD	0.2 ± 0.1
24	742 ± 12	120 ± 3	0.3 ± 0.1
25	521 ± 18	550 ± 5	3.3 ± 0.4
26	314 ± 21	170 ± 7	1.8 ± 0.3
27	127 ± 13	270 ± 3	2.4 ± 0.5
28	648 ± 9	380 ± 12	2.2 ± 0.2
29	765 ± 9	250 ± 21	2 ± 1
30	537 ± 8	520 ± 5	3.6 ± 0.9
31	983 ± 21	570 ± 19	3 ± 1
32	197 ± 13	78 ± 12	1.7 ± 0.3
33	253 ± 7	LOD	0.3 ± 0.1
34	585 ± 23	LOD	0.3 ± 0.1
35	730 ± 13	LOD	0.3 ± 0.1
36	639 ± 17	130 ± 13	0.3 ± 0.1
37	790 ± 24	450 ± 12	3.1 ± 0.7

## 4 Conclusion

Metal cycles in the environment are associated with the food chain: soil–plant–animal–man. So, the transfer of toxic metals to the higher link results in a cumulative increase in their available concentrations. Also, the chemistry of metal cycles determines their existence, speciation and contact itineraries to motherly acquisition and transmission to the breastfed newborns. Human breast milk samples were observed to contain the selected essential and non-essential (toxic and trace) metals in various concentrations. The detection of metals in the breast milk samples might result from various factors and sources such as environment and food. However, this is not an advocacy for formula feeding which is neither free of possible contamination. Routine sampling and analysing of breastfeeding milk is necessary to identify and minimize exposure pathways of metal contamination to the breastfed. This would thereby create awareness for the breastfeeding mothers to monitor their feeding habits and food consumption lifestyles. The concerned medicals should advocate for breastfeeding milk screening programme.

## Significance statement

This study discovered and reported for the first time in Nigerian lactating mothers’ breastmilk rare earth element Rb. This study will help researchers to focus more on rare earth elements and possible impact in breastmilk of lactating mothers as well as toxic metals like Cd and Hg.

## CRediT authorship contribution statement

OO supervised the study, wrote the proposal, conducted the statistical analysis, wrote the initial draft. AD, AS and OO collected the breast milk samples and completed the questionnaires. AT, OS and WG conducted the chemical analysis and performed the laboratory work. All the authors read, commented on and approved the final manuscript.

## Declaration of Competing Interest

The authors declare that they have no known competing financial interests or personal relationships that could have appeared to influence the work reported in this paper.

## Data Availability

Data will be made available on request.
